# The Use of Gamification in the Self-Management of Patients With Chronic Diseases: Scoping Review

**DOI:** 10.2196/39019

**Published:** 2023-12-22

**Authors:** Xiting Huang, Xinyue Xiang, Yang Liu, Zhiqian Wang, Zhili Jiang, Lihua Huang

**Affiliations:** 1 Nursing Department The First Affiliated Hospital Zhejiang University School of Medicine Hangzhou China; 2 Department of Respiratory Medicine The First Affiliated Hospital, Zhejiang University School of Medicine Hangzhou China

**Keywords:** gamification, chronic diseases, self-management, scoping review, mobile phone

## Abstract

**Background:**

Chronic disease self-management is a public health issue of worldwide concern, and gamification is an emerging strategy to improve patients’ participation in chronic disease self-management. Some studies have summarized designs for the gamification of chronic disease self-management from the perspective of eHealth technology, but they have not mentioned differences in design methods, functions, and evaluation methods of gamified designs for self-management in different chronic diseases.

**Objective:**

This scoping review aims to synthesize the characteristics of realization forms, functions, and evaluation methods in chronic disease self-management gamification to improve self-management among the chronic disease population.

**Methods:**

We applied a methodological framework for scoping reviews and the PRISMA-ScR (Preferred Reporting Items for Systematic Reviews and Meta-Analyses extension for Scoping Reviews) checklist. As of January 7, 2023, we systematically searched 9 databases for relevant studies from January 2012 to December 2022. Related data were extracted based on the research questions. We calculated the frequencies, charted the quantitative data, and coded the extracted material for qualitative content analysis.

**Results:**

We retrieved 16,221 records, of which 70 (0.43%) met the eligibility criteria. In the included research, the target populations for gamified designs for self-management of chronic diseases included patients with stroke, cancer, diabetes, chronic obstructive pulmonary disease, coronary heart disease, obesity, and hypertension. Almost all studies mentioned technical support for gamification (68/70, 97%), mainly in the form of active video games (58/70, 83%); however, less than half of the studies mentioned the theoretical basis for gamification (31/70, 44%). There were 37 concepts or theories relevant to gamification design, most of which were in the field of psychology or were cross-disciplinary (n=33, 89%). Gamification for the self-management of chronic diseases has been widely recognized, including for promoting physical exercise and rehabilitation training (48/99, 48%), increasing initiative for symptom management (18/99, 18%), providing psychological support (14/99, 14%), improving cognitive function (12/99, 12%), and improving medication adherence (7/99, 7%). A total of 39 studies mentioned the gamification effect; however, we did not find a unified evaluation standard.

**Conclusions:**

This scoping review focuses on gamification designs for chronic disease self-management and summarizes the realization forms and functions of gamification in self-management for different patient populations. With practice in a gamified internet-based environment, patients can not only master the knowledge and skills of self-management in fascinating scenarios but also benefit from gaming experience and make better health-related decisions in real life. It is worth noting that a comprehensive evaluation of the users as well as a personalized and targeted intervention should be developed before gamification.

## Introduction

### Background

Most chronic diseases have obvious behavioral risk factors including smoking, physical inactivity, and unhealthy diet [1]. Therefore, the treatment and management of these diseases increasingly involve cooperation with patients, relying on self-management. Barlow et al [2] defined self-management as the ability to manage symptoms, treatment, physical and psychosocial changes as well as lifestyle changes that patients develop in coping with chronic diseases. However, poor self-management compliance of patients with chronic diseases, such as nonprescribed medication, lack of regular physical activity, and unhealthy eating habits, is a common clinical problem worldwide [3,4]. People with chronic diseases not only cannot see the benefits of self-management in the short term but also endure repeated and monotonous training or changes in past health habits, which is a struggle between low impulses and high cognitive control [5]. With the rapid development of technology, mobile health (mHealth) and telemedicine based on the internet and mobile electronic devices can provide internet-based health services for acute and chronic care, intervention, counseling, and social support for patients with chronic diseases [6]. The process of motivating individuals to find joy in monotonous, repetitive, or uninteresting tasks in a planned, selective way and guiding individuals to make positive changes is called “gamification.”

Gamification refers to the application of game design elements to nongame contexts [7]. The specific practice is to integrate game design elements such as points, badges, leaderboards, and avatars into nongame contexts such as marketing [8] and classroom teaching [9] to realize “meaningful play,” that is, to achieve serious purposes other than entertainment in an interesting process [10]. When gamification emerges in the field of health care, its serious purposes include, but are not limited to, helping patients with chronic diseases adhere to self-management [11], promoting their basic needs of autonomy, and balancing the unidirectional and paternalistic relationships between doctors and patients [12]. The core of gamification design lies in human-computer interaction and instant feedback, which conforms to the abovementioned difficulties and pain points in the self-management of patients with chronic diseases and is an important measure to break the failure of patients with chronic diseases to adhere to long-term self-management.

Since 2011, there has been an intense discussion about gamification, and the literature on gamification in the health care field has grown rapidly. In the field of rehabilitation and behavioral sciences, gamification attracts patients with cardiovascular disease [13-15], diabetes [16,17], and stroke [18-20] to participate more actively in the management of their long-term health problems with the application of mHealth, virtual reality (VR) technology, and robots.

### Objectives

Gamification in the field of chronic disease self-management is at an emerging stage. However, in some studies on gamification design in the field of health care, there is a lack of understanding of gamification as a design strategy, narrowly limiting its application to the use of video games [21] or blindly adding game design elements solely to encourage certain behaviors (such as physical activity) [22]. At present, the theoretical framework design of gamification is a hot topic [23]. Therefore, based on the evidence-based methodologies and from the perspective of gamification design, we integrated the forms, functions, and effect evaluation of gamification in the self-management of patients with chronic diseases to explore the mechanics, dynamics, and experience of gamification design of users with chronic diseases.

Considering the heterogeneity of scientific research design and measurement methods of different studies, we believe that a scoping review is a method of knowledge synthesis that is suitable for discussing large-scale, diverse, and complex heterogeneity themes. For these reasons, a scoping review could be a better choice than a systematic review to quickly describe the research progress of a certain field, showing the scope, depth, breadth, and deficiency and providing more information for the future [24]. Therefore, this scoping review aimed to classify, compare, and synthesize gamification in chronic disease self-management.

## Methods

### Overall Framework

This study was designed according to the framework developed by Arksey and O’Malley [25]. The five main stages are as follows: (1) identify the research question (RQ), (2) identify relevant studies, (3) search strategy and study selection, (4) chart the data, and (5) summarize and report the results. This scoping review is reported according to the PRISMA-ScR (Preferred Reporting Items for Systematic Reviews and Meta-Analyses extension for Scoping Reviews) checklist.

### Research Question

This scoping review aimed to describe how gamification design has been implemented in self-management promotion for patients with chronic diseases and how to evaluate the effect of gamification in the self-management of patients with chronic diseases. We articulated the study objectives using the following RQs:

- RQ1: How is the gamification of chronic disease self-management implemented?
- RQ2: What role does gamification play in the self-management of chronic diseases?
- RQ3: How do existing studies evaluate the gamification design of chronic disease self-management?

### Eligibility Criteria of Relevant Studies

The research team used brainstorming to establish the eligibility criteria for relevant studies. According to the definition of chronic disease by the Centers for Disease Control and Prevention, we retrieved synonyms for chronic disease and specific disease names for cardiovascular diseases, chronic pulmonary diseases, metabolic syndrome, and cancer. On the basis of the widely accepted definition of gamification [7], relevant studies should apply games or game design elements to promote self-management needs with joyful experiences of patients with chronic diseases. We retrieved cognates and synonyms of gamification, game based, game design elements, game component, game design, and game principle. Given the inseparable connection between the application of gamification and electronic devices, we also retrieved synonyms of video games, computer games, exergames, and VR.

### Information Sources and Search Strategy

PubMed, Web of Science, CINAHL, APA PsycINFO, Embase, Cochrane Library, China National Knowledge Network, Wanfang (Wanfang Data), and VIP (CQVIP) were used as the data sources. In the past decade, there has been an increase in health-oriented gamified clinical research and applications [11]. Considering the speed of electronic technology changes, the technology before 2012 does not have a reference value, and we searched for the literature on gamification of chronic disease self-management from January 2012 to December 2022.

The retrieval strategy was based on the combination of subject terms and keywords, and the retrieval scope was the titles and abstracts. The search strategy did not limit any inclusion based on the affiliation of the authors, but the publication language was limited to English and Chinese to ensure an accurate understanding of the research. All databases were run on March 18, 2022, and updated on January 7, 2023. Refer to Multimedia Appendix 1 for the detailed search strategy.

### Inclusion and Exclusion Criteria

Under the guidance of an academic librarian, along with a brainstorming session, the research team set the following inclusion and exclusion criteria.

Inclusion criteria were as follows: (1) the research participants or targets were patients diagnosed with chronic diseases aged >18 years; (2) the gamification design in this study must include game design elements; and (3) the study was published in Chinese or English.

Exclusion criteria were as follows: (1) patients with diseases that require long-term rehabilitation owing to trauma (eg, patients with fractures or brain injuries); (2) special research participants such as high-risk groups of people with chronic diseases; groups people with genetic diseases; groups of people with occupational diseases; and special groups such as those who are disabled, sexual minorities, and pregnant women; (3) conference abstracts, patents for an invention, and so on, which only have descriptions but no experimental data; (4) studies for which the full texts are not available.

### Synthesis of Results

In total, 2 reviewers (XH and XX) independently screened abstracts and full-text articles with EndNote (version X9; Thomson Corporation) software. In case of conflicting opinions, a third reviewer was consulted (YL) to reach a consensus. The following information was extracted from the articles included in the review: first author, country, publication year, types of study design, objective population (chronic disease type), (if any) sample size and gender distribution, gamification realization forms, theoretical frameworks, game design elements, roles of gamification in chronic disease self-management, and gamification design evaluation tools. All data are presented with data charts suggested by Arksey and O’Malley [25].

To describe the sample and study characteristics, the first author coded papers for the following information using Excel (version 2019; Microsoft Corporation) software: publication year and country of publication (extracted from the paper metadata); types of study designs (extracted from the Methods section); number of patients with chronic diseases and their disease types (extracted from the Results section); and sample characteristics (extracted directly from the Results section or calculated mean value of all sample data).

To describe the application of gamification in chronic disease self-management, the first author coded the paper for following factors: gamification realization (extracted the description of the research tool from the Methods section); gamification evaluation (extracted related outcome measurements from the Methods section); gamification function (extracted verbatim from the “aim” or “objective” statements of each paper).

After data extraction, the fourth author calculated the frequencies of the quantitative data and charted the data. The first author coded the extracted material for qualitative content analysis. After 2 coding cycles, subthemes of “gamification forms” emerged, including “technical support” and “theoretical support,” and subthemes of “gamification function” emerged, including “symptom management,” “medication management,” “physical activity and rehabilitation,” “cognitive function rehabilitation,” and “psychological support.”

## Results

### Search Results

**Figure 1:**
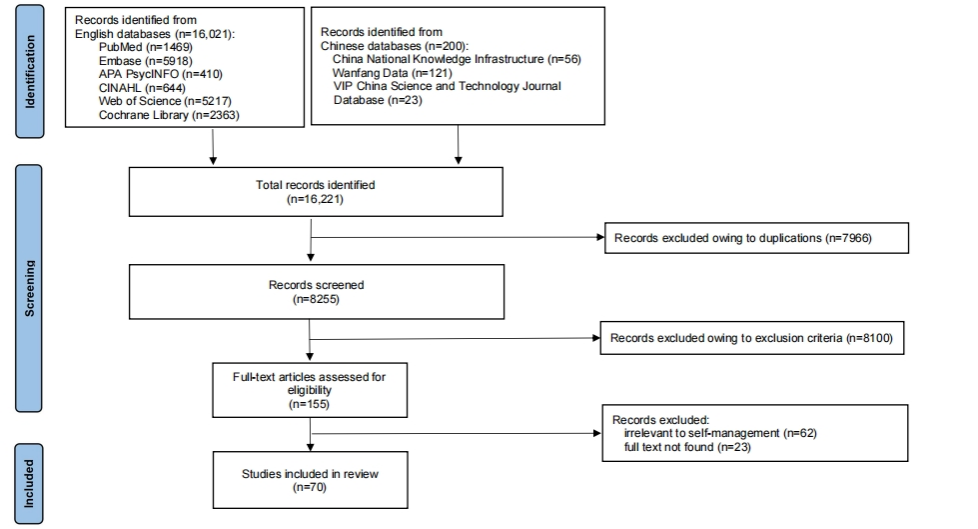
PRISMA (Preferred Reporting Items for Systematic Reviews and Meta-Analyses) flow diagram of study selection.

The initial search yielded 16,221 results, of which 7966 (49.11%) were duplicates and 8100 (49.94%) were excluded by reading the titles and abstracts. Of the remaining 155 articles, 70 (45.2%) met the inclusion criteria. The PRISMA-ScR flowchart (Figure 1) shows more detailed information [26].

### Basic Information

A total of 70 articles included in this review were mainly from 22 countries. Most studies (56/70, 80%) were concentrated in developed countries. The United States was the country with the most research (18/70, 26%), followed by the United Kingdom (6/70, 9%). There were 5 relevant studies in Canada and Australia and 3 relevant studies in China, Norway, Germany, Spain, Brazil, and Switzerland. The remaining 18 studies were conducted in 12 countries (South Korea, Greece, Iran, France, Malaysia, Portugal, Pakistan, Denmark, Japan, Singapore, Israel, and Indonesia).

Among the included studies, studies within the last 3 years accounted for half of the included studies (35/70, 50%). There were no restrictions on the study designs or chronic diseases in this review. The study design included experimental studies (27/70, 39%), literature studies (18/70, 26%), observational studies (13/70, 19%), and other types of studies (12/70, 17%). Survivors of stroke accounted for the largest number of studies (29/70, 41%), followed by patients with unclassified or multiple chronic diseases (11/70, 16%; Table 1). Except for some studies that only reported the development of gamification designs without data on research participants (26/70, 37%), the review extracted data from 2193 participants, most of whom were female (n=1423, 64.89%). The age distribution of the patients ranged from 33 to 85 years, with the mean age of 55.56 (SD 8.69) years (26/70, 37%). Details are provided in Multimedia Appendix 2 [13-20,27-87].

**Table 1:** Basic characteristics of the included studies (N=70).

Characteristic				Studies, n (%)
**Publication year**				
	2012-2015		9 (13)	
	2016-2019		26 (37)	
	2020-2022		35 (50)	
**Study design**				
	**Experimental study**		27 (39)	
		Randomized controlled trial	10 (14)	
		Pilot study	10 (14)	
		Mixed research	5 (7)	
		Quasi-experiment	1 (1)	
		Paired experiment design	1 (1)	
	**Literature study**		18 (26)	
		Systematic review	11 (16)	
		Scoping review	4 (6)	
		Review	3 (4)	
	**Observational study**		13 (19)	
		Qualitative research	9 (13)	
		Cross-sectional descriptive research	3 (4)	
		Retrospective research	1 (1)	
	**Other studies**		12 (17)	
		Introduction of the gamification design	6 (9)	
		Expert testimony	6 (9)	
**Patient population**				
	Hypertension		2 (3)	
	Obesity		2 (3)	
	Coronary heart disease		3 (4)	
	Chronic obstructive pulmonary disease		6 (9)	
	Diabetes		8 (11)	
	Cancer		9 (13)	
	Unclassified or multiple diseases		11 (16)	
	Stroke		29 (41)	

### Realization Forms of Gamification in Chronic Disease Self-Management

#### Overview

In this study, the gamification of chronic disease self-management cannot be achieved without advanced electronic and information technology but also requires a design supported by the theoretical model.

#### Technical Support

In this review, the gamification practice of chronic diseases requires the joint support of software (such as applications, active video games, websites, and emails) and hardware (game joysticks, computers, electronic displays, robots, and VR technology devices).

Owing to the derivation of gamification from games, gamification is a methodological approach that enables active video games (AVGs) to be given a purpose other than entertainment. In this study, AVGs were the most common form of gamification practice (58/70, 83%). AVGs are video games that require players to use a certain part of their body as a controller to interact with the game system [13,27]. They can be used as apps for smartphones, tablets, and desktops as well as in combination with hardware facilities such as game joysticks, electronic displays, robots, and VR technology to present AVG content. The top 3 diseases with the highest use rate for AVGs are stroke (28/58, 48%), cancer (8/58, 14%), and unclassified or multiple chronic diseases (7/58, 12%).

As AVGs rely on users’ activities in human-computer interaction, most studies (41/58, 71%) mentioned that users can physically interact with avatars in games through various physical activities, such as jogging and boxing, to promote physical activity and extremity function rehabilitation. According to whether AVGs are designed to serve the health care field, AVGs can be divided into commercial games for the general population and experimental games for patients with chronic diseases. The former ones aim for commercial profit, and their rule design and game operation do not have any therapeutic purposes, which has become a limitation in this type of clinical research [28]. In total, 4 qualitative interviews mentioned the gamification needs and preferences of patients with chronic diseases. Patients paid more attention to the health benefits of gamification than AVG points [29,30]. They hoped to integrate more social mechanisms to gain company from relatives and friends in the self-management process [31]. For older patients with chronic diseases, the AVG interface should be as simple as possible, and sound along with video can be used to replace excessive text and button functions [32]. One expert suggested that AVG design for stroke can use a storyline with a sense of purpose to alleviate the impact of significant physical and psychological changes after stroke, thus encouraging patients to participate in rehabilitation [33].

In addition, many hardware or software match (10/70, 14%) with communication media and integrate the game design elements to realize gamification of chronic disease self-management. The form of gamification of “hardware plus communication media” is specifically manifested as a wearable activity tracker combined with email and SMS text messaging [34,35] to provide feedback on exercise steps, which is used for postdischarge activities of patients with cancer [34], exercise, and weight loss for patients with diabetes [35]. The gamification forms of “software+communication media” include web-based interactive community websites [36], self-made mobile apps [37], and mHealth [14,16,38-41], which play an important role in knowledge education and symptom management of chronic obstructive pulmonary disease (COPD), diabetes, hypertension, and other diseases. In a qualitative interview, middle-aged and older patients with chronic diseases were asked how they felt about game design elements. They believe that the first encouragement from family and friends will determine their internal motivation for self-management, and the appropriate frequency of badges, likes, and other reward elements can motivate them to change their daily life habits [40]. Some experts have pointed out that different game design elements are interrelated; therefore, it is necessary to combine the characteristics of patients with their health and well-being and create a relatively stress-free game environment. Let patients enter the state of flow and improve the self-efficacy of self-management.

#### Theoretical Support

In this review, some studies (31/70, 44%) indicated that relevant theories or concepts guided their gamification practices in the self-management of patients with chronic diseases. A total of 37 concepts or theoretical frameworks were mentioned in the included articles, most of which originated from psychology and cross-disciplinarity (n=33, 89%), such as self-determination theory [38,40,42-44], flow theory [33,45-49], social cognition theory [27,50-52], goal-setting theory [34,43], information-motivation-behavioral technology model of behavior change [32,43], intrinsic motivation [16,53], self-efficacy [17,45], persuasion system design model [54], inhibitory control [55,56], patient-reported outcome [32], co-design approach, and participatory design [57].

Individual studies have started with game design, regarding gamification as a design strategy, and have developed frameworks, such as “mechanics-dynamics-aesthetics” frameworks [33,58], “mechanics-dynamics-emotions” frameworks [23], and “dynamics-mechanics-components” framework [88]. As the theoretical research of gamification is in an emerging direction, the terms are constantly changing.

In the abovementioned theoretical frameworks, the mechanics dimension is central to what defines a game, representing the rules, purpose, and scope of gameplay. The mechanics dimension is the fundamental process that drives game progress and player engagement, such as challenge mechanism [32,58], random mechanism [15,38,59], collaboration mechanism [15,31,37,59,89], and win-or-lose state. In contrast to the concept of mechanics, the dynamics dimension is not a concrete element directly applied to gamification design but rather an abstract motivation that promotes the achievement goals of targeted behavior. The dynamics dimension defines the nature of player interaction within a game, which gives rise to a change in the game state, both in the underlying data and the game’s interface. Common dynamics dimension in gamification design includes emotion (evoking players’ curiosity, excitement, happiness, or competitive motivation), narration (starting with a series of storylines), progression (recording the development and growth of players), and relationships (promoting family affection, friendship, and other emotions generated in the interaction) [89]. The aesthetics dimension emphasizes the relationship between a player’s game experience and their emotional or intellectual response to the game experience. In the included articles, graphics, audio, and haptics can provide sensory feedback and a fun experience for patients with chronic diseases, helping to promote the self-efficacy and response-efficacy of chronic disease self-management [90]. Along with users interacting with the gamified system, they will have emotional responses such as excitement, regret, pleasure, and thrill [23]. Some scholars have changed the word of “aesthetics” to the word of “emotion” (mechanics-dynamics-emotions) [23]. Each component of the gamification frameworks mentioned earlier is causally linked by the game designers and provides the users with pleasure and fun perceptions [91]. In the “dynamics-mechanics-components” framework, points, badges, and leaderboards were concrete game design elements, which only belong to the basic design of the component level in the gamification design. To achieve the maximum value of gamification, game design elements should be applied from the mechanical level preferably to the dynamic level [89].

Some studies have discussed the framework of gamification design in the self-management of patients with chronic diseases based on their gamification practice. “The wheel of Sukr” framework was proposed based on the self-management needs of people with diabetes, and the topic definition was summarized through expert interviews, providing practical guidelines for the gamification of chronic disease self-management. This theoretical framework combines behavior change methods and game technology with chronic disease self-management, emphasizing the close correlation among 8 themes: fun, esteem, growth, motivation, sustainability, socializing, self-representation, and self-management. “The wheel of Sukr” framework plays a synergistic role with the game design elements represented under each theme [60]. “The Ayogo model” was derived from the management evidence of patients with diabetes and established patients’ self-efficacy and belief in self-management through 3 mechanisms of “narrative,” “progression,” and “social interaction” [61].

In summary, the gamification design has achieved the purpose of promoting patients gain confidence in self-management through role-playing in games [58], encouraging patients to persist in rehabilitation with relatives or fellow patients [37,88], mastering disease knowledge and skills in an enjoyable and more engagement way [15,31]. For designers, gamification is about designing mechanics that create an aesthetic experience. However, for players, it is about having fun and then figuring out the rules and nonentertaining goals behind the form of game experience. Therefore, the gamification design of chronic disease self-management should be based on game design concepts, psychological or interdisciplinary terms, and immersive technologies such as VR and apply game design elements to provide users with a game experience to better support them in creating overall value in self-management.

### Roles of Gamification in Chronic Disease Self-Management

**Figure 2:**
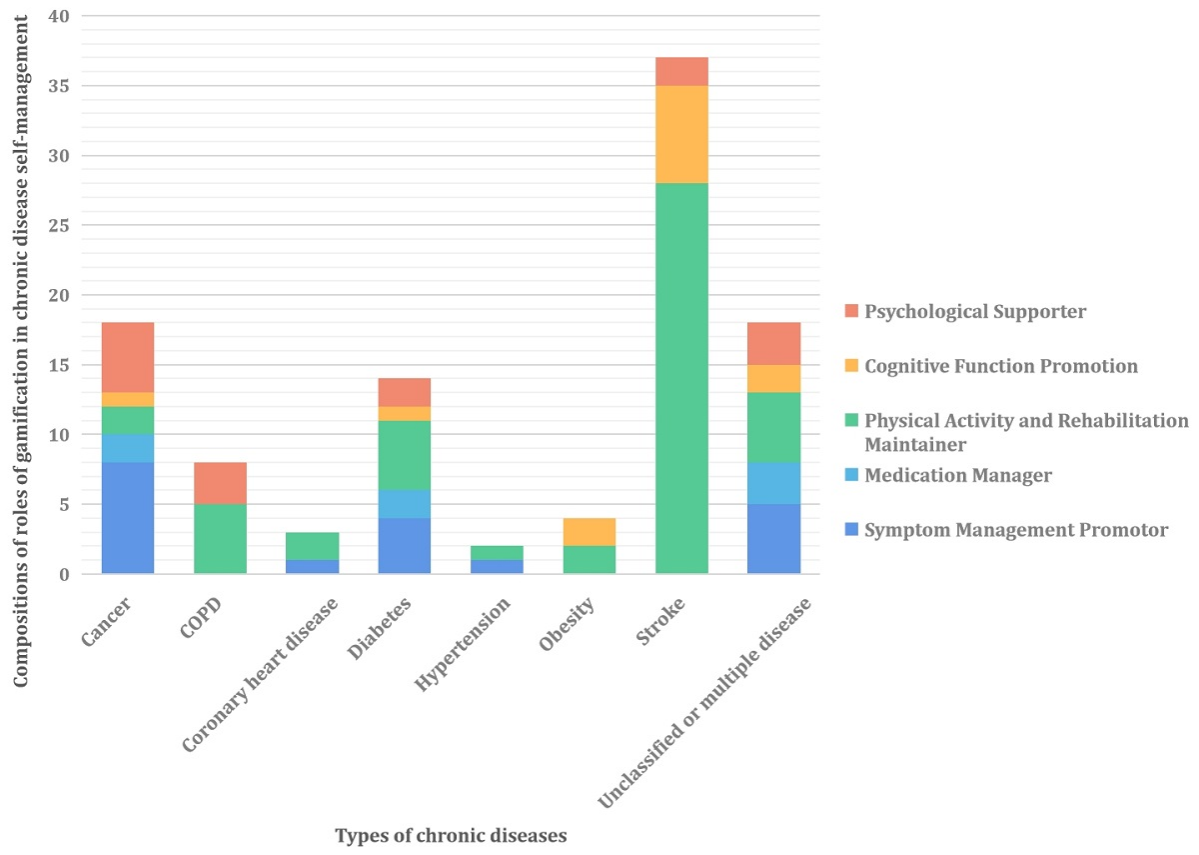
Roles of gamification in chronic disease self-management. COPD: chronic obstructive pulmonary disease.

Gamification in the self-management of patients with chronic diseases helped to manage symptoms, improve medication compliance, maintain physical activity and rehabilitation motivation, and provide psychological support (Figure 2).

#### Symptom Management Promotor

Within the scope of this review, cancer and diabetes management were prominent in gamification.

For patients with cancer, gamification enhanced the connection between patients with cancer and others through human-computer interaction and social mechanisms [29,32,34,45,51], providing support from health care professionals, relatives, and friends. The progress bar element was applied to record their health condition, self-management measures, and psychological progress [28,34,62], giving users a sense of disease control. In the management of side effects and symptoms after cancer chemotherapy, the project iManageCancer offered survivors of cancer an opportunity to play the role of a mayor with the avatar element. They could master strategies for handling chemotherapy-induced nausea and vomiting after chemotherapy by managing the “Anticancer City” in the game [62]. The avatar element enabled survivors of cancer to better immerse themselves in a virtual environment while playing games and mastering skills to relieve helplessness about chemotherapy-induced nausea and vomiting and other side effects after chemotherapy and alleviate the anxiety about death of survivors of cancer [51,63].

For people with diabetes, game design elements were applied for blood glucose management education. In a study that collected data on 14 gamification practices of diabetes self-management [89], the avatar element was mainly used to simulate insulin injection and diet management to maintain normal blood sugar levels. Take The Didget game for example, the source of AVG points was each reading of the glucose meter, users could exchange these points for game props, characters, and levels to promote timely measurement of blood glucose levels in people with diabetes [89]. It can internalize users’ behavior into personal goals during repeated training, improving their self-efficacy and promoting active participation in symptom management in the real world.

In symptom management, the avatar element enabled patients to learn more knowledge about disease management through role-playing [17,57,61-64]. Users were allowed to self-manage trials and errors in the virtual environment with an immersive experience, which cultivated the self-efficacy of self-management in repeated training. Finally, patients with chronic diseases should be guided to apply the disease management methods learned from the virtual environment in the real world and achieve the purpose of secondary prevention of disease.

#### Medication Manager

This review on medication adherence focused on survivors of cancer, patients with diabetes, and patients with other unclassified chronic diseases.

In the homemade mobile game iLoveBreast, the storyline element promoted patients with breast cancer to gradually complete self-education. The mean value of medication compliance of patients in the game group was higher than in the control group (7.6, SD 0.7 vs 6.5, SD 0.5; *P*<.001). This was because involvement in game medication and reminder alerts had improved users’ knowledge of the disease and medication safety [64].

For people with diabetes, insulin use had been taken as a routine [89], so even simple rewards (eg, cumulative points in the form of grades and badges) encouraged people with diabetes to practice glycemic control [40,89]. The time-limited mechanism was convenient for patients with diabetes to play repeatedly at their leisure and constantly consolidated the clinical diabetes education content in repeated processes [52,89].

Among other unclassified chronic diseases, a study retrospectively observed the compliance rate of medication time and dosage in patients with multiple chronic diseases who used the app (“Perx Health”) for 3 months and 6 months. This study found a slight downward trend in the 3-month group from the first month to the third month and in the 6-month group from month 4 to month 6 (*P*=.73) but found no significant differences in medication adherence rates over time (*P*>.05). In timing adherence subanalysis, the median medication adherence for the first month in the 6-month group was 77.3% (IQR 52.0%-93.1%) and 77.4% for the sixth month (IQR 36.2%-94.4%) [38]. This showed that gamification intervention can promote patients’ internal motivation and maintain long-term stability of medication adherence.

Although the number of studies on drug compliance in this review was limited, the results showed that medication education for patients with chronic diseases required a simple gamification design to avoid information overload [40,49]. However, it was necessary to inform the users when to terminate gamification intervention if the gamification design used the element of “points and levels” to record the growth of users. Unless users are intrinsically motivated to stick to medication, missing out on rewards could impair their compliance [38].

#### Physical Activity and Rehabilitation Maintainer

On the basis of Nintendo Wii series products, Kinect sensors, Microsoft Xbox system, and other mature somatosensory game products and research designs, there were a large number of studies (47/70, 52%) on physical activity and rehabilitation training, including patients with COPD [27,37,65-67], coronary heart disease [13,59,68], diabetes [16,28,35,89], cancers [34,51], and stroke [18-20,30,33,44,47-49,53,54,70-85], making physical activity and rehabilitation training enjoyable.

With the support of AVGs, patients with COPD could meet the exercise requirements of medium- to high-intensity resistance exercise [65,67], and the specific movements of the AVG could be regarded as a therapeutic exercise for patients with cardiovascular disease [13], which improved the compliance of pulmonary rehabilitation and cardiac rehabilitation [13,65,67]. With consideration of the characteristics of exercise intolerance of patients with COPD and coronary heart disease, safety for home use, different levels of difficulty and warm-up exercises were shown in some studies [66,68]. In physical activity interventions for people with diabetes, gamification design mainly promotes walking. Pokemon Go was a good example; its fascinating storyline and the user’s devotion to animation inspired the intrinsic motivation of users to use Pokemon Go, which attracted users to increase their steps, walking time, and training intensity during the 24-week intervention period [16]. Thus, gamification design was helpful in maintaining their physical activity, but it was difficult to completely replace therapeutic skills training such as pulmonary rehabilitation [66].

The gamification of rehabilitation for survivors of stroke was one of the breakthroughs in the field of health care, with a specific focus on the rehabilitation of upper limb function (22/70, 46%). For the rehabilitation of the upper arm and shoulder joint, motion sensors were worn to record the range of motion of the shoulder and the amount of shoulder movement, so as to help health care professionals and users judge in a timely manner whether the rehabilitation action was effective [20,30,47,69,70,73,76,78,83,85]. For hand function rehabilitation, game joystick, game gloves, and touch screen could be used to practice grasping, releasing, and pointing [53,71,73,78] while completing game tasks. For users with weak hand strength and small torso roll angles, commercial game devices cannot record subtle movement changes. Gloves with infrared technology [71] and tangible robots [18,20,44,80] emerged, capturing subtle movements invisible to the naked eye, providing timely feedback on every bit of rehabilitation progress to users, and empowering them to continue. As for functional rehabilitation of the lower limbs, gamification design focused on improving balance functions [42,69,74,84] and activity endurance [48,75,84]. In addition to AVGs, VR technology was combined to construct virtual scenes to train the spatial orientation and cognitive abilities of patients with stroke [18,49,73,74,82].

Overall, “human-computer interaction” was an important game design element in the gamification design of the physical activity and rehabilitation training, providing timely feedback data for both health care professionals and patients such as training duration, intensity, and training action; allowing patients to see physical function changes with exercise rehabilitation; and improving their compliance with exercise and rehabilitation [13,16,19,20,28,30,33,35,37,42,44,46-49,51-56,58-60,64-66,69-83,85].

#### Cognitive Function Promotion

The studies on cognitive function in the included studies were all conducted in the form of AVGs, which was applied to the attention training of obese patients and the rehabilitation of cognitive function after stroke and cancer chemotherapy.

AVGs come with the effect of cognitive function training. This is because the user learns the rules of the game and processes them quickly and accurately in the game, which keeps the brain active to avoid cognitive decline [45] and improves cognitive control and attention [42]. The time-limited game design element was used to train attention and goal-oriented behavior in obese patients, control the frequency of their consumption of a high-carbohydrate diet (eg, dessert), and strengthen their adherence to a healthy diet [55,56]. Users who received the gaming intervention were more concerned about daily food intake than those who received regular weight management (65.3% vs 33.6%), and the average weight loss in the intervention group was greater than that in the control group (3.4% vs 2.5%) [56].

On the basis of neuroplasticity, rehabilitation of cognitive impairment is possible for survivors after cancer chemotherapy or stroke [19,45,53,86]. A literature review on AVGs in older patients with chronic diseases mentioned that AVGs helped to improve executive functions such as naming, memory, and sense of direction [42]. One neurocognitive learning application was also effective in improving the speed and accuracy of information processing in survivors of cancer with chemotherapy-related cognitive impairment, and the effects could be sustained for some time (at the end of intervention: the average score of perceived cognitive impairment was −7.47, 95% CI −10.80 to −4.13; *P*<.001 and 6 months later: the average score was −6.48, 95% CI −9.85 to −3.11; *P*=.001) [45]. In terms of improving attention and perception, gamification design combined with VR could provide users with an immersive experience with the first-person perspective of game characters [19,49].

It is worth noting that technical interaction should avoid complex design for patients with cognitive dysfunction, which was preferably set as the default setting for self–decision-making (eg, group decision-making and social decision-making) [19,52,77,82,86]. It was also necessary to avoid eye fatigue caused by the long-term use of electronic screens and be aware of the uncanny valley effect of VR [33,72,73].

#### Psychological Supporter

Patients with chronic diseases usually experience loneliness [29,36], anxiety [33,41,46,63], depression [32,33,42,45,51,60,63,71,80,81], boredom, and pain [17,79] during the rehabilitation process. Some patients with chronic diseases tend to self-isolate [45]. Gamification design provided interesting experiences to distract patients with chronic diseases from negative emotions and provided support and encouragement from friends [14,27,35,37,77], other patients [36,37,44,53], and health care professionals [14,27,29,32,34,36,79].

Owing to the disease characteristics, survivors of cancer have a stronger fear of death than patients with other chronic diseases. They often focus on a vicious cycle of negative conditions and have difficulty getting rid of negative thoughts, leading to further anxiety and depression. One qualitative study mentioned that the points element was used to provide timely feedback on cumulative progression and improve motivation and positive emotions to disperse the fear of death, thus fundamentally shaking the foundation of negative emotions [32]. Another study introduced a web-based community platform (“COPD360 social”), allowing patients with COPD to exchange self-management experiences through the social mechanism and seek help from health care professionals. It enhanced the empowerment of patients with COPD and their caregivers, encouraged them to use the platform actively, created a sense of belonging in web-based communities, and promoted the dissemination of health knowledge [36].

The psychological effects of the gamification interventions were not independent. A systematic evaluation indicated that physical activity games were important components of the effective management of depression in the older adults, significantly reducing the degree of depression in older adults (SD mean difference=−0.60, 95% CI −0.95 to −0.25; *P*<.001) [87]. However, the psychological effects of gamification interventions were limited. If the patient had been diagnosed with depression, the intensity and frequency of the AVG-based intervention were not effective [42].

### Evaluation of Gamification Design in Chronic Diseases Self-Management

This review did not find a unified evaluation criteria for the gamification design of chronic disease self-management. The evaluation indicators of gamification design mentioned in the literature have been compiled into Table 2.

**Table 2:** Evaluation of gamification design (n=39).

Evaluative dimensions and evaluation methods		Studies, n (%)
**User satisfaction**		
	User Satisfaction Evaluation Questionnaire [78]	1 (3)
	Self-designed questionnaire [52,61,62]	3 (8)
	One-dimensional subjective score [35,51,55]	3 (8)
**User experience**		
	User Experience Questionnaire [58]	1 (3)
	Game Experience Questionnaire [53]	1 (3)
	Self-designed questionnaire [32,44,49,74,78,91]	6 (15)
	Interview [13,16,27,29-31,37,49,58,63,65,69,78,80]	14 (36)
**User preference**		
	User interface features [58]	1 (3)
	Self-designed questionnaire [41,77,78]	3 (8)
	Interview [13,40,57,58,73,78]	6 (15)
**Completion or participation**		
	Statistical results [55,56,62,68]	4 (10)
	Self-designed questionnaire [14]	1 (3)
**Accessibility**		
	Ease of Use Scale [32]	1 (3)
	Acceptance Questionnaire (based on Technology Acceptance Model) [14]	1 (3)
**Usability**		
	System Usability Scale [32,37,84]	3 (8)
**Feasibility**		
	Recruitment rate or retention rate [51,56]	2 (5)
**Safety**		
	Adverse events [35,68,73]	3 (8)
**Self-management behavior record**		
	System log files [14,16,19,20,44,47,65,69,81,91]	10 (26)
**Economic cost**		
	User Satisfaction Evaluation Questionnaire [78]	1 (3)

Of the 70 articles, 39 (56%) reported the evaluation indicators of the chronic disease self-management gamification system. Of the 39 articles, 9 (23%) used existing scales, including the System Usability Scale [32,37,85], Ease of Use Scale [32], Acceptance Questionnaire [14], User Experience Questionnaire [58], User Interface Features Questionnaire [58], User Satisfaction Evaluation Questionnaire [80], and Game Experience Questionnaire [53]. Of the 39 articles, 12 (31%) used self-designed scales to evaluate users’ satisfaction [52,64,65], experience [32,44,49,63,76,80], engagement [14], and preference [41,79,80]. Of the 39 articles, 10 (26%) with gamification design through AVGs used system log files to record symptom management strategies [14,63] and their duration [16,20,59,71], activity and rehabilitation training intensity, and related data of users [19,44,47,83].

In total, 17 studies showed user experience and preferences through interviews.

Most users mentioned experiencing pleasure and effectiveness when using gamified systems [29,58,59,66,68,71,80,82]. They could receive timely feedback for error correction or affirmation with human-computer interaction [58,82], stimulate self-efficacy of self-management with the competition mechanism [49,57], and realize personal progress visualization and internal motivation promotion with game design elements such as points and the progress bar [40]. On the basis of mobile devices, users’ physical activities and rehabilitation training could be free from the limitations of time, location, weather, or other medical activities, achieving self-management liberalization and confidentiality [16,30,37,66,71]. Using the internet, users could obtain support and supervision from health care professionals and patients more conveniently [27,31,37]. However, some users were older adults, and obstacles in technology and health status were inevitable [75]. They stated that they were unfamiliar with electronic technology and required accompaniment or technical support from their family members or health care professionals [13,27,31,66,71,75]. Owing to the severity of the disease, individual differences in participation and challenge difficulty among different users made it difficult for the gamification design to achieve absolute fairness [27,57]. Therefore, the gamification design of the system interface required comprehensive consideration of users’ cognitive and physical functions. This could be simplified by reducing the number of buttons instead of background music to prevent visual information overload [49,57]. Security preparedness was the most important issue when AVG was used for physical activity or rehabilitation training. Without it, users would not have the motivation to use the gamification system [77]. It would be helpful to present action guidance from the first perspective in AVG so that users can operate better and increase their interest, thereby entering a flow state [49]. In terms of competition mechanism and goal setting of gamification design, user engagement and actual situations showed individual differences, preferably allowing users to set their own goals to avoid physical exhaustion and disrupting the fun of exercise itself [27,29].

Overall, the evaluation focused on the experience of patients with chronic diseases, reflecting the self-management motivation, technical novelty, fun of the game experience, and the degree of personalization related to the motivation for self-management [30,82]. Only a few studies reported safety-related outcomes [35,70,75] and adverse events triggered by individual exercises with a gamification system (such as knee pain and low back pain) [70]. We suggest that to promote gamification in chronic disease self-management, health care professionals should pay attention to the safety protection and emergency plan for patients’ home use.

## Discussion

### Principal Findings

This scoping review identified 70 gamification studies on chronic disease self-management conducted from 2012 to 2022. Stroke was the leading disease in gamification research (29/70, 41%), and the field of physical activity and functional rehabilitation was the central research content of chronic disease self-management gamification. In all studies, gamification practice was dominated by AVGs (58/70, 83%), and its theoretical support was mainly derived from existing psychological and cross-disciplinarity concepts or theories. The main functions of gamification were promoting physical exercise and rehabilitation training in patients with chronic diseases (48/99, 48%), especially in the field of poststroke rehabilitation; AVGs have become a rehabilitation tool for survivors of stroke [48] and are used for symptom management (18/99, 18%), psychological support (14/99, 14%), cognitive exercise (12/99, 12%), and medication management (7/99, 7%). Evaluation indicators and qualitative text showed that the use of gamification in chronic disease self-management was widely recognized by patients.

### Reflection

Compared with traditional self-management methods, the combination of gamification design and mobile technology has broken through the traditional medical mechanism and experience of patients with chronic diseases, allowing them to have more autonomy in the choice of rehabilitation training time and rehabilitation forms. Therefore, gamification enabled patients with chronic diseases to maintain a high motivation for self-management for 6 months [38], meeting the requirements of disease self-management such as physical activity, symptom management, and disease cognition. In addition, patients with chronic diseases who coexisted with diseases for a long time often had negative emotions such as anxiety and depression, which had a negative impact on their health outcomes, disease management, and quality of life [87]. Gamification used game design elements to create enjoyable experiences, allowing patients to better immerse themselves and complete self-management in a state of flow [46,57,73]. When patients reacted as the requirements of self-management, the gamification would provide timely praise through human-computer interaction, like points accumulation. This timely positive feedback helped to improve the self-efficacy of patients with chronic diseases engaged in self-management, and they sincerely felt happy [79], thereby reducing the conflict between long-term health goals and previous unhealthy habits [50,60], fostering sustainable long-term self-management.

We suggest that health care professionals, developers, and researchers of gamification design should take ethnic characteristics, personal lifestyles, personality, interests, and hobbies into consideration when applying gamification. The most controversial game design element in this study was the competitive mechanism. Competition is bound to result in winners and losers. It is undoubtedly an incentive for patients who win and lead the leaderboard, but if the patient is far behind others, it may make the user give up [57]. This could be a disease characteristic of the susceptibility to fatigue. Patients with cancer and patients with COPD believe that game design elements should be used to build confidence, and even if they want to compete, they should constantly challenge and surpass themselves, rather than emphasizing the competition for patient performance. Patients with different disease severity levels have different perceptions of difficulty in completing the same challenge. For those with severe disease, failure to challenge, especially over a long period, may even lead to depression and a lack of self-efficacy [27,29,45]. For people with diabetes, the fun of competition lies in the fast-paced disease knowledge competition [52,89]. Therefore, the application of game design elements should be related to the frequency of interactions between users and the system or the degree of participation between users and the community to gamify the experience of chronic disease self-management. In addition, patients with chronic diseases of different ages have different preferences for game design elements. Among middle-aged and older adults, users do not want too many rewarding elements such as badges and likes, avoiding making the gamification system of chronic disease self-management like a children’s game [40]. Technical barriers should be considered when older patients with chronic diseases use electronic products, which reminds the gamification designer to simplify the game interface and game rules without affecting the user’s fun [46,49,52,58,61,77,79].

Therefore, successful gamification design cannot be separated from the following four universal gamification design rules: (1) focus on incentive mechanism, (2) do not terminate games at will, (3) not overemphasizing specific gamification mechanism, and (4) link games with health and happiness [39]. Before the gamification design of chronic disease management, 4 core questions are recommended to be based on the following: Can it provide a good motivation for chronic disease self-management? Is the set target activity meaningful and interesting? Is there a structured pattern of the expected activity behavior? Does the gamification mechanism use potentially conflict with the target behavior?

### Future Research

Although this review found that gamification had a positive and universal effect on chronic disease self-management, we still need to view the results dialectically. Most of the gamification intervention periods in the included studies were all within 1 year, and most interventions were within 6 months; therefore, we cannot rule out the novelty effect of technology. We also need to consider that the users included are themselves interested in the study, which inevitably leads to a selection bias. In addition, the gamification of chronic disease self-management involved multiple environments such as clinical settings [73,80] and family settings [30,44,77], which interferes with the effectiveness of gamification interventions. To better explore how gamification promotes self-management in patients with chronic diseases and promotes their expected long-term outcomes, the consistency of the research setting should be controlled, the sample size should be expanded, and the follow-up period should be extended in the future, and more methodological research is encouraged to investigate the extent to which various forms of gamification impact chronic disease self-management.

### Limitations

Unfortunately, this review retrieved only the literature published in Chinese or English, omitting valuable data from relevant articles published in other languages. Gray material and unpublished studies were not included in this review. Many health-related applications were privately operated, and their development and evaluation information were not disclosed. The sample sizes of some diseases in the included studies were insufficient and could not represent the entire population. Therefore, in the future, we will expand the search to focus on the literature published in other languages and further study the heterogeneity of disease populations.

### Conclusions

This review was based on a definition of gamification, without limiting the type of research, that focused on gamification designs for chronic disease self-management; the study synthesized the differences in the gamification realization forms and functions in self-management of different disease populations, which helped the researchers to completely understand the use experience and opinions on gamification for the self-management of patients with chronic diseases. Before using gamified interventions for chronic disease self-management, researchers must conduct a comprehensive survey of the target group, clarify the shortcomings of the self-management and game preferences of patients with chronic diseases, and stimulate internal motivation through appropriate game design elements. At the same time, it is also necessary to consider the multidisciplinary expert qualifications of the research team and the cost of research interventions, developing attractive chronic disease self-management plans based on the definition of gamification to be applicable to chronic disease management in developing countries.

## Appendix

Multimedia Appendix 1 Searching strategies of this scoping review.

Multimedia Appendix 2 Data extraction of the references.

## Abbreviations

AVG: active video game
COPD: chronic obstructive pulmonary disease
mHealth: mobile health
PRISMA-ScR: Preferred Reporting Items for Systematic Reviews and Meta-Analyses extension for Scoping Reviews
RQ: research question
VR: virtual reality

## Data Availability

All data generated or analyzed during this study are included in this paper and the multimedia appendices.
