# Supplementary material for: The Use of Gamification in the Self-Management of Patients With Chronic Diseases: Scoping Review
Source: JMIR Serious Games. 2023 Dec 22;11:e39019. doi: 10.2196/39019 (PMC10770795; doi:10.2196/39019)
Supplement: Multimedia Appendix 1 [file games_v11i1e39019_app1.docx]

Searching Strategies

**PubMed:**

("Chronic Disease"[Mesh] OR "Noncommunicable Diseases"[Mesh] OR "chronic illness"[Title/Abstract] OR "chronic condition"[Title/Abstract] OR "noninfectious disease*"[Title/Abstract] OR "Cardiovascular Diseases"[Mesh] OR "Hypertension"[Mesh] OR "High Blood Pressure"[Title/Abstract] OR "Coronary Disease"[Mesh] OR "Coronary Artery Disease"[Mesh] OR "heart valve disease*"[Title/Abstract] OR "heart disease*"[Title/Abstract] OR "Stroke"[Mesh] OR "Cerebrovascular Accident"[Title/Abstract] OR "Pulmonary Disease, Chronic Obstructive"[Mesh] OR "Asthma-Chronic Obstructive Pulmonary Disease Overlap Syndrome"[Mesh] OR "Asthma"[Mesh] OR "COPD"[Title/Abstract] OR "Chronic obstructive pulmonary disease"[Title/Abstract] OR "Chronic Obstructive Lung Disease"[Title/Abstract] OR "Chronic Bronchitis"[Title/Abstract] OR "Pulmonary Arterial Hypertension"[Title/Abstract] OR "Pulmonary Heart Disease"[Title/Abstract] OR "Metabolic Syndrome"[Mesh] OR "Diabetes Mellitus"[Mesh] OR "Diabetes Mellitus, Type 1"[Mesh] OR "Diabetes Mellitus, Type 2"[Mesh] OR "Obesity"[Mesh] OR "Diabetes"[Title/Abstract] OR "Neoplasms"[Mesh] OR "tumor*"[Title/Abstract] OR "cancer*"[Title/Abstract] OR "malignant tumor*"[Title/Abstract]) AND ("Gamification"[Mesh] OR "Video Games"[Mesh] OR "Exergaming"[Mesh] OR "Virtual Reality"[Mesh] OR "Virtual Reality Exposure Therapy"[Mesh] OR "gamif*"[Title/Abstract] OR "computer game*"[Title/Abstract] OR "active video game*"[Title/Abstract] OR "exergam*"[Title/Abstract] OR "game-based"[Title/Abstract] OR "game design*"[Title/Abstract] OR "game element*"[Title/Abstract] OR "game component*"[Title/Abstract] OR "game principle*"[Title/Abstract] OR "playful elements"[Title/Abstract]) AND (2012:2022[pdat]) AND (chinese[Filter] OR english[Filter])

**Web of Science:**

(TS=(Chronic Disease) OR TS=(Noncommunicable Diseases) OR TS=(Chronic Illness) OR TS=(Chronic Condition) OR TS=(noninfectious disease*) OR TS=(Cardiovascular Diseases) OR TS=(Hypertension) OR TS=(High Blood Pressure) OR TS=(Coronary Disease) OR TS=(Coronary Artery Disease) OR TS=(Heart Valve Disease*) OR TS=(heart disease*) OR TS=(Stroke) OR TS=(Cerebrovascular Accident) OR TS=(Asthma-Chronic Obstructive Pulmonary Disease Overlap Syndrome) OR TS=(Asthma) OR TS=(COPD) OR TS=(Chronic obstructive pulmonary disease) OR TS=(Chronic Obstructive Lung Disease) OR TS=(Chronic Bronchitis) OR TS=(Pulmonary Arterial Hypertension) OR TS=(Pulmonary Heart Disease) OR TS=(Metabolic Syndrome) OR TS=(Diabetes Mellitus) OR TS=(Diabetes type 1) OR TS=(Diabetes type 2) OR TS=(Obesity) OR TS=(Diabetes) OR TS=(Neoplasms) OR TS=(tumor*) OR TS=(cancer*) OR TS=(malignant tumor*)) AND (TS=(Video Game*) OR TS=(Active Video Game*) OR TS=(Exergaming) OR TS=(Virtual Reality) OR TS=(Virtual Reality Exposure Therapy) OR TS=(gamif*) OR TS=(computer game*) OR TS=(exergam*) OR TS=(game-based) OR TS=(game design*) OR TS=(game element*) OR TS=(game component*) OR TS=(game principle*) OR TS=(playful element*)) AND (LA==("ENGLISH" OR "CHINESE"))

Filter: Published Date= 2012-01-01 - 2022-12-31

**CINAHL:**

SU ("chronic disease*" OR "noncommunicable disease*" OR "chronic illnesses" OR "chronic condition*" OR "noninfectious disease*" OR "Cardiovascular Diseases" OR "Hypertension" OR "High Blood Pressure" OR "Coronary Disease" OR "Coronary Artery Disease" OR "Heart Valve Disease*" OR "heart disease*" OR "Stroke" OR "Cerebrovascular Accident" OR "Asthma-Chronic Obstructive Pulmonary Disease Overlap Syndrome" OR "Asthma" OR "COPD" OR "Chronic Obstructive Pulmonary Disease" OR "Chronic Obstructive Lung Disease" OR "Chronic Bronchitis" OR "Pulmonary Arterial Hypertension" OR "Pulmonary Heart Disease" OR "Metabolic Syndrome" OR "Diabetes Mellitus" OR "Diabetes type 1" OR "Diabetes type 2" OR "Obesity" OR "Diabetes" OR "Neoplasm" OR "tumor*" OR "cancer*" OR "malignant tumor*") AND SU ("Gamification" OR "gamif*" OR "Video Game*" OR "Active video game*"OR "Exergaming" OR"exergam*" OR "Virtual Reality" OR "Virtual Reality Exposure Therapy" OR "computer game*" OR "game-based" OR "game design*" OR "game element*" OR "game component*" OR "game principle*" OR "playful element*")

Filter: Language=English OR Chinese ; Published Date=2012-01-01 - 2022-12-31

**APA PsycInfo:**

SU ("chronic disease*" OR "noncommunicable disease*" OR "chronic illnesses" OR "chronic condition*" OR "noninfectious disease*" OR "Cardiovascular Diseases" OR "Hypertension" OR "High Blood Pressure" OR "Coronary Disease" OR "Coronary Artery Disease" OR "Heart Valve Disease*" OR "heart disease*" OR "Stroke" OR "Cerebrovascular Accident" OR "Asthma-Chronic Obstructive Pulmonary Disease Overlap Syndrome" OR "Asthma" OR "COPD" OR "Chronic Obstructive Pulmonary Disease" OR "Chronic Obstructive Lung Disease" OR "Chronic Bronchitis" OR "Pulmonary Arterial Hypertension" OR "Pulmonary Heart Disease" OR "Metabolic Syndrome" OR "Diabetes Mellitus" OR "Diabetes type 1" OR "Diabetes type 2" OR "Obesity" OR "Diabetes" OR "Neoplasm" OR "tumor*" OR "cancer*" OR "malignant tumor*") AND SU ("Gamification" OR "gamif*" OR "Video Game*" OR "Active video game*"OR "Exergaming" OR"exergam*" OR "Virtual Reality" OR "Virtual Reality Exposure Therapy" OR "computer game*" OR "game-based" OR "game design*" OR "game element*" OR "game component*" OR "game principle*" OR "playful element*")

Filter: Language=English; Published Date=2012-01-01 - 2022-12-31

**Embase:**

(('chronic disease'/exp OR 'non communicable disease'/exp OR 'cardiovascular disease'/exp OR 'hypertension'/exp OR 'coronary artery disease'/exp OR 'heart disease'/exp OR 'cerebrovascular accident'/exp OR 'asthma-chronic obstructive pulmonary disease overlap syndrome'/exp OR 'asthma'/exp OR 'chronic obstructive lung disease'/exp OR 'chronic obstructive lung disease'/exp OR 'chronic bronchitis'/exp OR 'metabolic disorder'/exp OR 'diabetes mellitus'/exp OR 'insulin dependent diabetes mellitus'/exp OR 'non insulin dependent diabetes mellitus'/exp OR 'obesity'/exp OR 'neoplasm'/exp) OR (('chronic condition' OR 'noninfectious disease' OR 'High Blood Pressure' OR 'coronary disease' OR 'Heart Valve Disease*' OR 'stroke' OR 'Pulmonary Heart Disease' OR 'Diabetes type 1' OR 'Diabetes type 2' OR 'Diabetes' OR 'tumor*' OR 'cancer*' OR 'malignant tumor*'):ti,ab)) AND (('gamification'/exp OR 'video game'/exp OR 'virtual reality'/exp OR 'exergaming'/exp) OR ('exergame' OR 'Virtual Reality Exposure Therapy' OR 'gamif*' OR 'computer game*' OR 'active video game*' OR 'game-based' OR 'game design*' OR 'game element*' OR 'game component*' OR 'game principle*' OR 'playful element*'):ti,ab)

Filter: Language=English OR Chinese ; Published Date=01-01-2012 - 31-12-2022

**Cochrane Library：**

Title Abstract Keyword ("chronic disease*" OR "noncommunicable disease*" OR "chronic illnesses" OR "chronic condition*" OR "noninfectious disease*" OR "Cardiovascular Diseases" OR "Hypertension" OR "High Blood Pressure" OR "Coronary Disease" OR "Coronary Artery Disease" OR "Heart Valve Disease*" OR "heart disease*" OR "Stroke" OR "Cerebrovascular Accident" OR "Asthma-Chronic Obstructive Pulmonary Disease Overlap Syndrome" OR "Asthma" OR "COPD" OR "Chronic Obstructive Pulmonary Disease" OR "Chronic Obstructive Lung Disease" OR "Chronic Bronchitis" OR "Pulmonary Arterial Hypertension" OR "Pulmonary Heart Disease" OR "Metabolic Syndrome" OR "Diabetes Mellitus" OR "Diabetes type 1" OR "Diabetes type 2" OR "Obesity" OR "Diabetes" OR "Neoplasm" OR "tumor*" OR "cancer*" OR "malignant tumor*") AND Title Abstract Keyword ("Gamification" OR "gamif*" OR "Video Game*" OR "Active video game*"OR "Exergaming" OR"exergam*" OR "Virtual Reality" OR "Virtual Reality Exposure Therapy" OR "computer game*" OR "game-based" OR "game design*" OR "game element*" OR "game component*" OR "game principle*" OR "playful element*")

Cochrane Library publication date Between Jan 2012 and Dec 2022, in Cochrane Reviews (Word variations have been searched)

**CNKI:**

(SU=('慢性病'+'慢性非传染性疾病'+'慢性消耗性疾病') OR TKA=('心脑血管疾病'+'心血管疾病'+'高血压'+'冠心病'+'高脂血症'+'动脉粥样硬化'+'脑卒中'+'卒中'+'中风'+'心脑血管疾病'+'心脑血管意外'+'慢性心律失常'+'心脏瓣膜疾病'+'心脏病'+'慢性呼吸系统疾病'+'慢性阻塞性肺疾病'+'COPD'+'慢阻肺'+'老慢支'+'慢性阻塞性肺气肿'+'支气管扩张'+'慢性支气管炎'+'哮喘'+'支气管哮喘'+'慢性肺炎'+'肺动脉高压'+'肺源性心脏病'+'肺心病'+'慢性肝炎'+'肝硬化'+'代谢综合征'+'肥胖症'+'糖尿病'+'瘤'+'癌'+'恶性肿瘤')) AND SU%=('游戏化'+'交互视频游戏'+'视频游戏'+'健身游戏化'+'健身游戏'+'运动游戏'+'虚拟游戏'+'模拟游戏'+'游戏设计元素')

Publish date：20120101-20221231

**Wanfang Database:**

（主题:（慢性病or慢性非传染性疾病or慢性消耗性疾病) or（题名或关键词:(心脑血管疾病or心血管疾病or高血压or冠心病or高脂血症or动脉粥样硬化or脑卒中or中风or心脑血管疾病or心脑血管意外or慢性心律失常or心脏瓣膜疾病or心脏病or慢性阻塞性肺疾病or COPD or慢阻肺or老慢支or慢性阻塞性肺气肿or支气管扩张or慢性支气管炎or哮喘or支气管哮喘or慢性肺炎or肺动脉高压or肺源性心脏病or肺心病or慢性肝炎or肝硬化or代谢综合征or肥胖症or糖尿病or瘤or癌or恶性肿瘤)) and (主题:(游戏化) or（题名或关键词:(视频游戏or交互视频游戏or健身游戏化or健身游戏or运动游戏or虚拟游戏or模拟游戏or 游戏设计元素))

Publish date：20120101-20221231

**CQVIP:**

题名或关键词:（（慢性病or慢性非传染性疾病or慢性消耗性疾病or心脑血管疾病or心血管疾病or高血压or冠心病or高脂血症or动脉粥样硬化or脑卒中or中风or心脑血管疾病or心脑血管意外or慢性心律失常or心脏瓣膜疾病or心脏病or慢性阻塞性肺疾病or COPD or慢阻肺or老慢支or慢性阻塞性肺气肿or支气管扩张or慢性支气管炎or哮喘or支气管哮喘or慢性肺炎or肺动脉高压or肺源性心脏病or肺心病or慢性肝炎or肝硬化or代谢综合征or肥胖症or糖尿病or瘤or癌or恶性肿瘤) and (游戏化or视频游戏or交互视频游戏or健身游戏化or健身游戏or运动游戏or虚拟游戏or模拟游戏or 游戏设计元素））

Publish year：2012-2022
